# Myocardial infarction models in NOD/*Scid* mice for cell therapy research: permanent ischemia vs ischemia–reperfusion

**DOI:** 10.1186/s40064-015-1128-y

**Published:** 2015-07-10

**Authors:** Vanessa-Leigh van Zuylen, Melina C den Haan, Helene Roelofs, Willem E Fibbe, Martin J Schalij, Douwe E Atsma

**Affiliations:** Department of Cardiology, Leiden University Medical Center, Albinusdreef 2, P.O. Box 9600, 2300 RC Leiden, The Netherlands; Department of Immunohematology and Blood Transfusion, Leiden University Medical Center, Albinusdreef 2, P.O. Box 9600, 2300 RC Leiden, The Netherlands

**Keywords:** Myocardial infarction, Ischemia/reperfusion, Inflammatory response, Cytokines, Cell therapy, Immunodeficient mice

## Abstract

Myocardial infarction animal studies are used to study disease mechanisms and new treatment options. Typically, myocardial infarction (MI) is induced by permanent occlusion of the left anterior descending artery. Since in MI patients coronary blood flow is often restored new experimental models better reflecting clinical practice are needed. Here, permanent ischemia MI (PI group) was compared with transient ischemia (45 min) (IR group) in immunodeficient NOD/*Scid* mice. Cardiac function, infarct size, wall thickness and total collagen deposition were significantly reduced only in PI mice. Cardiac inflammatory cells and serum cytokine levels were less dynamic in IR animals compared to PI. So although IR better reflects clinical practice, it is secondary to PI for investigating cell therapy, since it induces too little damage to provide a measurable therapeutic window. MI did result in significant changes in the inflammatory state, indicating this immunodeficient mouse strain is valuable to study human cell therapy.

**Electronic supplementary material:**

The online version of this article (doi:10.1186/s40064-015-1128-y) contains supplementary material, which is available to authorized users.

## Background

As cardiovascular diseases, including myocardial infarction (MI), remain one of the leading causes of death globally (Roger et al. 2012), the search for new therapies continues. Cell therapy has emerged over the past years as a potential treatment modality for MI patients in addition to conventional treatment (Zimmet et al. 2012). Before the various candidate cell types are to be applied in clinical trials, they have to be investigated in experimental disease animal models (Johnston et al. 2009; Williams and Hare 2011; Katare et al. 2011; Yoon et al. 2010; Gu et al. 2012). To mimic future clinical use of these cells as much as possible, typically human cells are studied in animal models. To avoid rejection of these infused cells by the immune system of the host and to avoid the use of pharmacological immune suppression, immune-deficient animals such as the non-obese diabetic/severe combined immunodeficient (NOD/*Scid*) mice strain are used. The *SCID* mutation results in an impaired T and B cell lymphocyte development which allows transplantation of different cell-types originating e.g. from peripheral blood, bone-marrow or organs (Simpson et al. 1991). MI in mouse models typically is induced through permanent ligation of the left anterior descending artery (LAD). However, in current clinical practice rapid restoration of coronary blood flow is the cornerstone of MI treatment, rendering the permanent coronary artery ligation mouse model less representative of the clinical situation.

Tissue inflammation is an important element in cardiac remodelling after MI. Coronary artery occlusion induces an inflammatory cascade which can be divided in three partially overlapping phases of inflammation, proliferation and maturation (Frangogiannis 2006). Reperfusion of infarcted cardiac tissue leads to an increased and accelerated inflammatory response possibly deteriorating cardiac remodelling (Entman and Smith 1994).

In the present study MI induced by permanent coronary occlusion of the LAD (PI group) was compared with ischemia–reperfusion MI by transient ligation of the LAD (IR group). MI was induced in the immunodeficient NOD/*Scid* mice, a strain necessary to study the effects of human cell therapy. In both MI models cardiac function was assessed by magnetic resonance imaging (MRI) and pressure–volume (PV) loop measurements. Furthermore infarct size was assessed by MRI and histology and parameters of local and systemic inflammatory response were investigated to determine the relevance of this immunodeficient mice strain in MI research.

## Methods

### Animals

Experiments were performed in 8- to 10-weeks-old male NOD/*Scid* mice (Charles River Laboratories, Maastricht, The Netherlands). Experiments were approved by the Committee on Animal Welfare of the Leiden University Medical Center and conformed to the *Guide for the Care and Use of Laboratory* as stated by the U.S. National Institutes of Health. Animals were housed in filter top cages and were given standard diet and water with antibiotics and antimycotics ad libitum.

### MI model

MI was induced in NOD/*Scid* mice by permanently (PI group) or transiently (45 min) (IR group) ligating the LAD. Sham-operated animals were used to determine baseline characteristics (Sham group). Mice received 100 µL NaCl, containing 2 µg buprenorphine, subcutaneously before surgery and again 12 h after surgery. The permanent ligation MI model was induced as described earlier (den Haan et al. 2012). Briefly, animals were anesthetized with 5% isoflurane for induction and kept anesthetized with 1.5–2% isoflurane in oxygen for the remainder of the surgical procedure, intubated and ventilated using a rodent ventilator (model 845, Harvard Apparatus, Holliston, MA, USA) with 160 breaths per min and a stroke volume of 220 μL. A left thoracotomy was performed and the LAD was ligated using a 7-0-prolene suture (Johnson and Johnson, New Brunswick, NJ, USA). For the permanent ligation experiment (PI group), the location of the LAD ligation was 1 mm caudally from the tip of the left auricle. For the transient ligation and subsequent reperfusion experiment (IR group), the LAD was transiently ligated for 45 min directly underneath the tip of the left auricle to induce maximum damage, followed by reperfusion. To allow reperfusion of the LAD after 45 min, the ligation was fixed on a tube placed directly on the LAD. Ischemia was confirmed by myocardial blanching. The chest was left open during the entire ischemic period. Based on pilot data transient ligation was performed for 45 min, since an ischemia duration ≥50 min resulted in unacceptable high mortality rates (100%, either during the ischemic period or afterwards within the first week of the procedure). Five min after LAD ligation in the PI group or 5 min after reperfusion in the IR group, animals received 15 μL phosphate-buffered saline intramyocardially to mimic intramyocardial cell therapy. After 35 min of LAD ligation mice received an intraperitoneal injection of lidocaine (6 mg/kg) to prevent cardiac arrhythmias (Tarnavski et al. 2004). Afterwards, the chest was closed and animals were allowed to recover. Sham animals were operated in parallel, but without LAD ligation and intramyocardial injection, and were used to determine baseline characteristics (Sham group).

The mortality rate of the IR group was 33.3% at the end of the experiment, with 25% mortality during the ischemic period or afterwards at the same day of the procedure and 75% mortality during the follow up period of the experiment or during MRI measurements.

The mortality rate of the PI group was 41.2% at the end of the experiment, with 14.3% mortality during the procedure or afterwards at the same day of the procedure and 85.7% mortality during the follow up period of the experiment or during MRI measurements.

The mortality rate of the Sham group was 25% at the end of the experiment with 100% mortality during the follow up period of the experiment or during MRI measurements.

### Cardiac MRI

Cardiac parameters were assessed 2 and 14 days post-MI using a 7-Tesla MRI (BrukerBiospin, Ettlingen, Germany). Mice were anesthetized with 5% isoflurane for induction and kept anesthetized with 1.5–2% isoflurane in oxygen for the remainder of the procedure and placed on a respiration detection cushion connected to a gating module to monitor respiratory rate (SA Instruments, Inc., Stony Brook, NY, USA). Image reconstruction was performed using BrukerParaVision5.1 software (BrukerBiospin, Ettlingen, Germany). To evaluate cardiac function, a high-resolution 2D FLASH cine sequence was used to acquire a set of 9 contiguous 1 mm slices in short-axis orientation covering the entire heart. Imaging parameters were: echo time of 1.49 ms, repetition time of 5.16 ms, field of view (26 mm)^2^ and a matrix size of 144 × 192. To determine infarct size, contrast enhanced MRI imaging was performed after injection of a 150 µL bolus (0.5 mmol/mL) of gadolinium-DPTA (Gd-DPTA, Dotarem, Guerbet, The Netherlands) via the tail vein. A gradient echo sequence (FLASH) was used to acquire a set of 14 contiguous 0.7 mm contract-enhanced slices in short-axis orientation covering the entire heart. Imaging parameters were: echo time of 1.9 ms, repetition time of 84.16 ms, field of view (33 mm)^2^ and a matrix size of 192 × 256. All MR data were analysed with the MASS for Mice software package (MEDIS, Leiden, The Netherlands). Endocardial and epicardial borders were manually delineated and a reference point was positioned by an investigator blinded to the experimental status of the data (PI group n = 5, IR group n = 5, Sham group n = 5, overall 15 mice for statistical analysis).

### Left ventricular function by PV loops

At day 15 after MI, mice were anesthetized again with 5% isoflurane for induction and kept anesthetized with 1–1.5% isoflurane in oxygen for the remainder of the procedure. Via the right carotid artery, a 1.2F pressure-conductance catheter (standard; ScisenseInc, London, Canada) was introduced and positioned in the left ventricle (LV). The conductance catheter was connected to a PV control unit FV 896B (ScisenseInc, London, Canada) for online display and recording of LV pressure and volume signals. Parallel conductance and LV pressure–volume signals were measured as described previously (den Haan et al. 2012; Steendijk and Baan 2000). All data were acquired using Powerlab 8/30 Model ML870 (ADInstruments, Spechbach, Germany) and LabChart 7 software (ADInstruments, Spechbach, Germany). Data were analyzed with custom-made software by an investigator blinded to the experimental status of the data (PI group n = 5, IR group n = 5, Sham group n = 5, overall 15 mice for statistical analysis).

### Histological infarct size

After PV loops hearts were dissected, fixed and cut as described earlier (PI group n = 4, IR group n = 4, Sham group n = 4, overall 12 mice for statistical analysis) (den Haan et al. 2012). The extent of total collagen deposition after MI was evaluated by staining with picro-sirius red (0.5 g Sirius red in 500 mL saturated aqueous solution of picric acid) for 1 h. Total collagen deposition was determined by the area stained tissue within the LV as a percentage of the whole LV. This analysis was performed on photomicrographs taken at a twofold magnification of 15–20 sections covering the entire heart from apex to base. The area of Sirius red staining was measured by an observer blinded to the experimental status of the samples, using the ImageJ software package (National Institutes of Health, Bethesda, MA, USA).

The effect of MI on LV wall thickness was assessed on the same sections. This analysis was performed on three equidistant sections between the apex and ligature (at the midpoint between the LAD ligature and the apex, between the midpoint and the LAD ligature and between the midpoint and apex). Wall thickness was measured at two separate border zone areas, at the midpoint of the infarct region and averaged for all three measurements. Measurements were performed perpendicular to the infarcted wall.

### Cardiac inflammation response

In parallel to the above described experiment, MI was induced by PI or IR in mice that were sacrificed on days 1, 3, and 7 after MI (n = 3 mice per time point, overall PI group n = 9, overall IR group n = 9). Non-operated animals were used as controls to determine base line characteristics at 1 time point (Control group, n = 3, overall 21 mice for statistical analysis). Hearts were harvested in total, minced with fine scissors, and placed into a solution of 2% collagenase I^A^ (Sigma-Aldrich, Saint Louis, MO, USA) in PBS and shaken at 37°C for 1 h. The cell suspension was then triturated through a nylon mesh and centrifuged in PBS at 300*g* for 10 min at 4°C. Red blood cells in the cell pellet were lysed with lysis buffer, and the cells were washed in PBS and subsequently resuspended in medium containing IMDM (Lonza, Verviers, Belgium) supplemented with 2.5% fetal calf serum (FCS; Greiner Bio-one, Monroe, NC, USA) and Penicillin/Streptomycin (P/S; Invitrogen Corp., Paisley, UK). Total cardiac cell numbers were determined with a Sysmex cell counter (Sysmex America, Inc. Mundelein, Illinois, USA). The resulting single-cell suspensions were stained for flow cytometry with primary antibodies for 30 min at 4°C in darkness and the cells were washed with PBS/1% human Albumin (Sanquin, Leiden, The Netherlands) before analysis using a FACSCanto II (BD Biosciences, San Diego, CA, USA). The following antibodies were used: anti-CD90-APC, 53–2.1, -B220-APC, RA3-6B2, -CD49b-APC, DX5, -NK1.1-APC, PK136, -Ly-6G-APC, 1A8, CD11b-eFluor 450, M1/70, -F4/80-FITC, -CD11c-FITC, HL3, -I-A^b^ -FITC, AF6-120.1, -Ly-6C-PE, AL-21, -CD11c-PE, HL3 (All above antibodies are from BD Biosciences) and C1:A3-1 (ABD Serotec, Kidlington, UK). Monocytes were identified as CD11b high (CD90/B220/CD49b/NK1.1/Ly-6G) low. They were further divided into Ly-6C high/low (F4/80/I-A^b^/CD11c) low as previously described (Nahrendorf et al. 2007; Swirski et al. 2009). Macrophages were identified as CD11b high, F4/80 high. Dendritic cells were identified as CD11b, I-A^b^ and CD11c high. Neutrophils were identified as CD11b, Ly-6G high. Monocyte and macrophage/dendritic cell numbers were calculated as the total cells multiplied by the percentage of cells within the monocyte/macrophage gate. The analysis of the acquired data was done with FlowJo software version 7.6.1 (Tree Star Inc. Ashland, OR, USA).

### Cytokine measurements

Cytokine measurements were performed on peripheral blood from the same mice that were used to determine the cardiac inflammation response. Mice were sacrificed on days 1, 3, and 7 after MI (n = 3 mice per time point, overall PI group n = 9, overall IR group n = 9). Non-operated animals were used as controls to determine base line characteristics (Control group, n = 3, overall 21 mice for statistical analysis). Peripheral blood was drawn via cardiac puncture and collected in eppendorf tubes to let the blood coagulate >2 h at room temperature. Subsequently, the samples were centrifuged to separate the blood clot from the serum and the serum was stored at −80°C until cytokine determination. Cytokine concentrations were measured using the Bio-PlexMouse Cytokine 23-plex Panel assay (Bio-Rad laboratories, Inc, Hercules, CA, USA).

### Statistical analysis

Numerical values were expressed as mean ± standard deviation (SD). Comparison of MRI parameters between the PI, IR and Sham group was performed using two-way repeated-measures analysis of variance (ANOVA), with Bonferroni correction. Comparison of the remaining parameters between the PI, IR and Sham or Control group was performed using one-way ANOVA, with Bonferroni correction.

## Results

### MRI: infarct size and cardiac function

**Figure 1:**
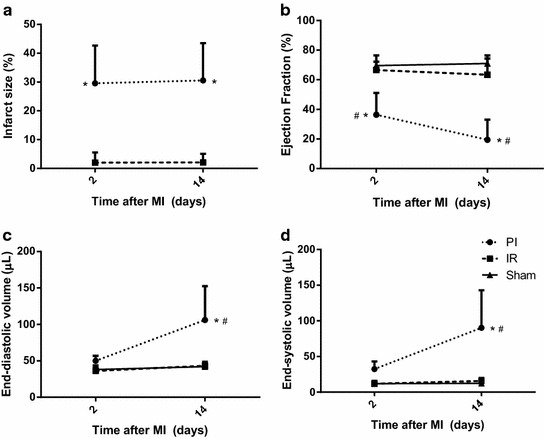
Assessment of left ventricular function and volumes with MRI. MRI analysis of delayed contrast-enhanced images showed a significant difference in infarct size (**a**) between the PI and IR group, both at t = 2 and t = 14. Assessment of left ventricular function of the PI, IR and Sham group at t = 2 and t = 14: ejection fraction (**b**), end-diastolic volume (**c**) and end-systolic volume (**d**). N = 5 per group. Data are expressed as mean ± SD. *p < 0.05 versus IR, ^#^p < 0.05 versus Sham.

Both 2 and 14 days after MI, the infarct size in animals that were subjected to PI (n = 5) was significantly larger than in the IR model (n = 5) (Figure 1a). This difference in damage between both MI groups was reflected by the cardiac function in the two models. Two days after MI, ejection fraction decreased significantly in the PI group, with a further deterioration of function 14 days after MI (Figure 1b). Both end-diastolic and end-systolic volumes tended to increase in the PI group 2 days post-MI and reached significance at day 14 after MI, when compared to the IR and Sham group (Figure 1c, d). Likewise, stroke volume, cardiac output, wall thickening and wall motion deteriorated significantly in PI animals at day 14 days after MI (Additional file 1: Table S1). In contrast, cardiac function parameters in IR animals did not significantly differ from those in Sham animals.

In accordance with the MRI data, PV loop measurements 15 days after MI showed a significant deterioration in cardiac function in the PI group, but not in the IR group (Additional file 2: Table S2).

### Histology: infarct size

**Figure 2:**
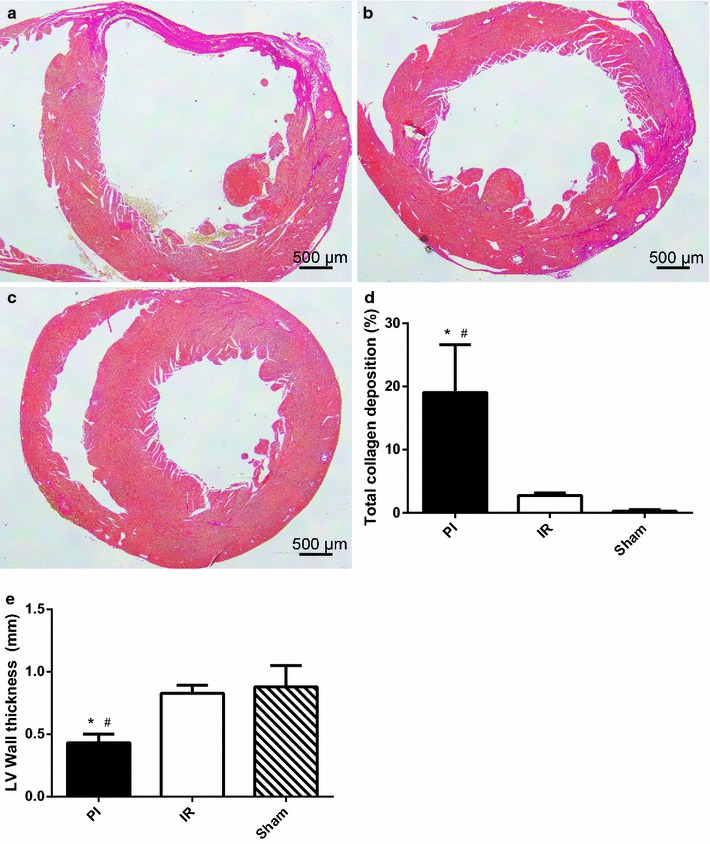
Histological quantification of total collagen deposition and wall thickness. Photos of representative sections of the hearts of PI (**a**), IR (**b**) and Sham (**c**) mice 15 days after MI showing total collagen deposition (*red*) by Sirius red staining. Quantification of Sirius red staining (**d**). Quantification of LV wall thickness (**e**). N = 4 per group. Data are expressed as mean ± SD. *p < 0.05 versus IR, ^#^p < 0.05 versus Sham.

Consistent with the observed difference in infarct size observed by MRI, animals that were subjected to PI showed a significant increase in total collagen deposition (19.0%) 15 days after MI, as opposed to IR (2.7%) and Sham (0.3%) (n=4 in all groups) (Figure 2a–d). In addition, quantification of wall thickness showed a significant thinning of the LV wall after MI in the PI group (0.43 mm), but not in the IR group (0.83 mm), where wall thickness was comparable to the Sham group (0.88 mm) (Figure 2e).

### Cardiac inflammation response

**Figure 3:**
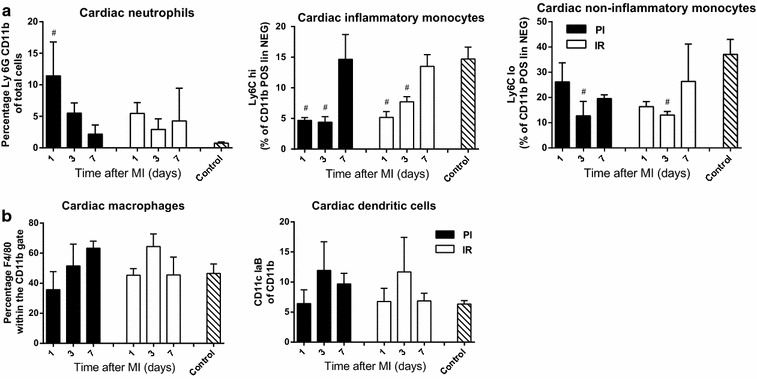
Flow cytometric analysis of inflammatory cells. MI in the PI model induces marked neutrophil recruitment and an egress of monocytes (**a**) within 7 days after injury measured via flow cytometry on hole heart digests. Cardiac macrophages and dendritic cell percentages are unaltered after myocardial injury (**b**). N = 3 per group. Data are expressed as mean ± SD. ^#^p < 0.05 versus Control.

The effect of MI on the local influx of inflammatory cells in both the PI and IR group was assessed by flow cytometry and compared to non-operated control animals. Flow cytometric analyses on single cell heart digests revealed significantly increased neutrophil frequencies in the PI group on the first day after MI, which declined thereafter (Figure 3a). Cardiac inflammatory monocyte populations however, were significantly reduced in both the PI and IR group at both day 1 and 3 after MI. The frequency of non-inflammatory monocytes was reduced as well in both groups, but the kinetic for this cell population was slower as it was only significant 3 days after MI.

Cardiac macrophages and dendritic cell frequencies in either of the MI groups, however, did not significantly deviate from the control group, suggesting these cell types are less relevant in the cardiac inflammatory process within the first week after MI (Figure 3b).

### Cytokine measurements

**Figure 4:**
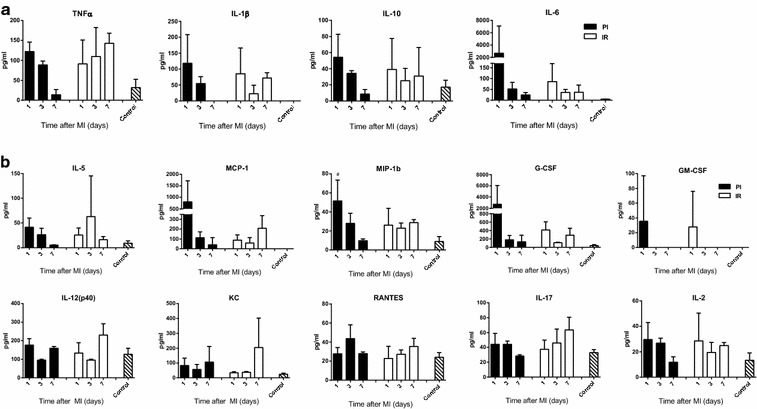
Cytokine levels in blood serum. Blood serum analyses appear to show a more dynamic induction in the PI group within 1 day after MI of TNFα, IL1β, IL-10, and IL-6, which are important cytokines after MI (**a**) and a range of various other cytokines (**b**). N = 3 per group. Data are expressed as mean ± SD. ^#^p < 0.05 versus Control.

In the PI group, the average serum levels of the known relevant pro-inflammatory cytokines tumor necrosis factor α (TNF-α), IL-1β, and IL-6, and the anti-inflammatory cytokine IL-10 were found to be increased at day 1 post MI, and these serum levels gradually decreased thereafter. Statistically, however these differences did not meet significance (Figure 4a). In the IR group injury induced lower levels of these cytokines but these seemed to be sustained for a longer period. In addition, the other cytokines that were measured via ELISA were IL-5, MCP-1, MIP-1β, G-CSF, GM-CSF, IL-12(p40), KC, RANTES, IL-17, and IL-2, of which MCP-1, MIP-1β, G-CSF appeared to be more dynamic in the PI group compared to the IR group, although this was not significantly different (Figure 4b).

## Discussion

Key findings of the current study are that MI by IR results in (1) a non-significant deterioration of cardiac function and remodeling compared with Sham animals, (2) a reduced population of cardiac inflammatory cells, (3) limited dynamics in serum cytokine levels and (4) non-significant total collagen deposition and wall thinning.

So, despite the resemblance of the IR model to the current clinical practice, this study demonstrates its role for cell therapy optimization is limited, since IR fails to inflict sufficient myocardial damage to create the experimental window necessary to monitor significant improvement.

Earlier research by Vandervelde et al. already showed less pronounced remodelling in mouse hearts subjected to transient ligation, when compared to permanent ligation (Vandervelde et al. 2006). A more recent study, which compared transient ligation to permanent ligation by cryoinfarction showed the same results (Duerr et al. 2011). However, it should be noted that permanent ligation by cryoinfarction is mechanistically different to permanent ligation used in this study. Permanent ischemia by cryoinfarction resulted in a prolonged remodelling period and excess remodelling, when compared to transient ligation (Duerr et al. 2011). Although cardiac function was not investigated in both studies, the possible difficulties in testing a therapeutic intervention due to less damage were discussed.

Our findings on infarct size differences between the PI and IR group were consistent with other studies, which also showed a significantly larger infarct size after permanent ligation, measured by both contrast-enhanced MRI (Luo et al. 2012) and histological staining (Luo et al. 2012; Michael et al. 1999). Furthermore, we found that only PI resulted in significant thinning of the LV wall, which was also in line with earlier research (Vandervelde et al. 2006). Solely based on the small decline in cardiac function and the limited cardiac remodelling after MI, IR induces little damage, while ischemia durations ≥50 min resulted in unacceptable high mortality rates in a pilot study. The mostly observed cause of death in this pilot study was death by cardiac arrhythmia, which was monitored by 3-lead EKG (data not shown). Overall, IR leaves a small window for therapeutic intervention.

Besides the effect of PI and IR on cardiac function and remodeling, we also investigated and compared the inflammatory response. After occlusion of a coronary artery several chemokine and cytokine cascades are activated during the first phase of cardiac inflammation, the inflammatory phase. Subsequently dead cells and matrix debris are removed from the wound by neutrophils and macrophages. This is followed by the deposition of extracellular matrix proteins and formation of an extensive microvascular network during the proliferation phase of cardiac inflammation. Afterwards, maturation of the scar follows and a collagen-based scar is formed (Frangogiannis 2006, 2008) during the final maturation phase.

After MI inflammatory monocytes present in the cardiac tissue give rise to macrophages, while the role of non-inflammatory monocytes is not yet clear (Nahrendorf and Swirski 2013). In the current study the frequency of the inflammatory and non-inflammatory monocytes was reduced in both models within the first week after MI, suggesting the inflammatory monocytes are indeed differentiating into macrophages. However, no increased number of macrophages in the cardiac tissue in either the PI or the IR model was observed. In addition, a significant influx of neutrophils in the PI group was detected. This is in contrast to earlier studies by Luo et al. and Vandervelde et al. where increased influx of macrophages and neutrophils was observed after reperfusion compared to permanent ligation (Vandervelde et al. 2006; Luo et al. 2012). Also no increase in dendritic cell numbers was detected here, as opposed to observations in other experimental settings (Anzai et al. 2012).

In the current study, the upregulation of inflammatory cytokines after MI, initiating the inflammatory process, was modest compared to earlier research (Frangogiannis 2008; Moro et al. 2007). However, a direct comparison in cytokine levels between the PI and IR group suggests that the extent of injury dictates the systemic inflammatory response, since the limited cardiac damage in the IR model related to the less dynamic changes in the cytokine profile. The observed differences in inflammatory response between this study and earlier research may be explained by the difference in immune systems between the mice strains used. Since the primary goal of the current study was to investigate human cell therapy in a more clinically relevant MI model immunodeficient mice were used, which have an impaired repertoire of immune cells, capable of performing innate immunity only. Despite of this impaired immune response in NOD/*Scid* mice, this study shows that inducing MI still results in significant changes in the immune state of these animals, indicating this mouse strain can still be valuable to study human cell therapy.

## Conclusions

MI by transient ligation results in a limited loss of cardiac function, minimal cardiac remodeling and a less dynamic inflammatory response, compared to the most widely used model of permanent ligation. So, although a model of transient ligation may better mimic the clinical situation, the limited myocardial damage renders it less suitable to study novel therapies including stem cell therapy. Furthermore even in the immunodeficient mice strain used, MI does significantly alter the inflammatory state.

## Additional files

Additional file 1:
**Table S1.** MRI derived LV function indices for PI, IR and Sham-operated mice. Additional file 2:
**Table S2.** PV loops derived LV function indices for PI, IR and Sham-operated mice.

## References

[CR1] Anzai A, Anzai T, Nagai S, Maekawa Y, Naito K, Kaneko H (2012). Regulatory role of dendritic cells in postinfarction healing and left ventricular remodeling. Circulation.

[CR2] den Haan MC, Grauss RW, Smits AM, Winter EM, van TJ, Pijnappels DA (2012). Cardiomyogenic differentiation-independent improvement of cardiac function by human cardiomyocyte progenitor cell injection in ischaemic mouse hearts. J Cell Mol Med.

[CR3] Duerr GD, Elhafi N, Bostani T, Ellinger J, Swieny L, Kolobara E (2011). Comparison of myocardial remodeling between cryoinfarction and reperfused infarction in mice. J Biomed Biotechnol.

[CR4] Entman ML, Smith CW (1994). Postreperfusion inflammation: a model for reaction to injury in cardiovascular disease. Cardiovasc Res.

[CR5] Frangogiannis NG (2006). The mechanistic basis of infarct healing. Antioxid Redox Signal.

[CR6] Frangogiannis NG (2008). The immune system and cardiac repair. Pharmacol Res.

[CR7] Gu M, Nguyen PK, Lee AS, Xu D, Hu S, Plews JR (2012). Microfluidic single-cell analysis shows that porcine induced pluripotent stem cell-derived endothelial cells improve myocardial function by paracrine activation. Circ Res.

[CR8] Johnston PV, Sasano T, Mills K, Evers R, Lee ST, Smith RR (2009). Engraftment, differentiation, and functional benefits of autologous cardiosphere-derived cells in porcine ischemic cardiomyopathy. Circulation.

[CR9] Katare R, Riu F, Mitchell K, Gubernator M, Campagnolo P, Cui Y (2011). Transplantation of human pericyte progenitor cells improves the repair of infarcted heart through activation of an angiogenic program involving micro-RNA-132. Circ Res.

[CR10] Luo D, Yao YY, Li YF, Sheng ZL, Tang Y, Fang F (2012). Myocardial infarction quantification with late gadolinium-enhanced magnetic resonance imaging in rats using a 7-T scanner. Cardiovasc Pathol.

[CR11] Michael LH, Ballantyne CM, Zachariah JP, Gould KE, Pocius JS, Taffet GE (1999). Myocardial infarction and remodeling in mice: effect of reperfusion. Am J Physiol.

[CR12] Moro C, Jouan MG, Rakotovao A, Toufektsian MC, Ormezzano O, Nagy N (2007). Delayed expression of cytokines after reperfused myocardial infarction: possible trigger for cardiac dysfunction and ventricular remodeling. Am J Physiol Heart Circ Physiol.

[CR13] Nahrendorf M, Swirski FK (2013). Monocyte and macrophage heterogeneity in the heart. Circ Res.

[CR14] Nahrendorf M, Swirski FK, Aikawa E, Stangenberg L, Wurdinger T, Figueiredo JL (2007). The healing myocardium sequentially mobilizes two monocyte subsets with divergent and complementary functions. J Exp Med.

[CR15] Roger VL, Go AS, Lloyd-Jones DM, Benjamin EJ, Berry JD, Borden WB (2012). Heart disease and stroke statistics—2012 update: a report from the American Heart Association. Circulation.

[CR16] Simpson E, Farrant J, Chandler P (1991). Phenotypic and functional studies of human peripheral blood lymphocytes engrafted in scid mice. Immunol Rev.

[CR17] Steendijk P, Baan J (2000). Comparison of intravenous and pulmonary artery injections of hypertonic saline for the assessment of conductance catheter parallel conductance. Cardiovasc Res.

[CR18] Swirski FK, Nahrendorf M, Etzrodt M, Wildgruber M, Cortez-Retamozo V, Panizzi P (2009). Identification of splenic reservoir monocytes and their deployment to inflammatory sites. Science.

[CR19] Tarnavski O, McMullen JR, Schinke M, Nie Q, Kong S, Izumo S (2004). Mouse cardiac surgery: comprehensive techniques for the generation of mouse models of human diseases and their application for genomic studies. Physiol Genomics.

[CR20] Vandervelde S, van Amerongen MJ, Tio RA, Petersen AH, van Luyn MJ, Harmsen MC (2006). Increased inflammatory response and neovascularization in reperfused vs. non-reperfused murine myocardial infarction. Cardiovasc Pathol.

[CR21] Williams AR, Hare JM (2011). Mesenchymal stem cells: biology, pathophysiology, translational findings, and therapeutic implications for cardiac disease. Circ Res.

[CR22] Yoon CH, Koyanagi M, Iekushi K, Seeger F, Urbich C, Zeiher AM (2010). Mechanism of improved cardiac function after bone marrow mononuclear cell therapy: role of cardiovascular lineage commitment. Circulation.

[CR23] Zimmet H, Porapakkham P, Porapakkham P, Sata Y, Haas SJ, Itescu S (2012). Short- and long-term outcomes of intracoronary and endogenously mobilized bone marrow stem cells in the treatment of ST-segment elevation myocardial infarction: a meta-analysis of randomized control trials. Eur J Heart Fail.

