# Supplementary material for: Myocardial infarction models in NOD/Scid mice for cell therapy research: permanent ischemia vs ischemia–reperfusion
Source: Springerplus. 2015 Jul 10;4:336. doi: 10.1186/s40064-015-1128-y (PMC4498004; doi:10.1186/s40064-015-1128-y)
Supplement: Additional file 1: — Table S1. MRI derived LV function indices for PI, IR and Sham-operated mice. [file 40064_2015_1128_MOESM1_ESM.docx]

**Table 1. MRI derived LV function indices for PI, IR and Sham-operated mice.**

|  | **PI**  **T=2** | **T=2** | **IR**  **T=2** | **Sham**  **T=2** | **PI**  **T=14** | **T=14** | **IR**  **T=14** | **Sham**  **T=14** |
| --- | --- | --- | --- | --- | --- | --- | --- | --- |
| **SV, µL** | **20.9 ± 4.0** |  | **21.3 ± 2.8** | **25.2 ± 2.8** | **19.5 ± 4.9** | *, # | **27.6 ± 5.2** | **29.5 ± 1.8** |
| **CO, mL/min** | **14.3 ± 2.7** |  | **14.6 ± 1.9** | **17.2 ± 1.9** | **13.4 ± 3.4** | *, # | **18.9 ± 3.6** | **20.2 ± 1.3** |
| **ED wall thickness, mm** | **0.74 ± 0.04** |  | **0.74 ± 0.06** | **0.69 ± 0.06** | **0.60 ± 0.07** | # | **0.67 ± 0.08** | **0.70 ± 0.04** |
| **ES wall thickness, mm** | **0.99 ± 0.08** |  | **1.12 ± 0.10** | **1.06 ± 0.10** | **0.77 ± 0.11** | *, # | **1.09 ± 0.07** | **1.17 ± 0.05** |
| **Wall thickening, %** | **37.1 ± 8.7** | **#** | **54.4 ± 10.5** | **59.9 ± 12.0** | **29.5 ± 11.0** | *, # | **67.7 ± 20.5** | **70.2 ± 13.7** |
| **Wall motion, mm** | **0.46 ± 0.10** | ***, #** | **0.65 ± 0.11** | **0.68 ± 0.06** | **0.30 ± 0.11** | *, # | **0.72 ± 0.02** | **0.74 ± 0.05** |

Abbreviations: SV, stroke volume; CO, cardiac output; ED, end diastolic; ES, end systolic.
N=5 per group. Data are expressed as mean ± SD. * = p<0.05 versus IR; # = p<0.05 versus Sham
