# Supplementary material for: Myocardial infarction models in NOD/Scid mice for cell therapy research: permanent ischemia vs ischemia–reperfusion
Source: Springerplus. 2015 Jul 10;4:336. doi: 10.1186/s40064-015-1128-y (PMC4498004; doi:10.1186/s40064-015-1128-y)
Supplement: Additional file 2: — Table S2. PV loops derived LV function indices for PI, IR and Sham-operated mice. [file 40064_2015_1128_MOESM2_ESM.docx]

**Table 2. PV loops derived LV function indices for PI, IR and Sham-operated mice.**

|  | **PI** | **IR** | **Sham** | **P-value:  PI *versus* IR** | **P-value:  PI *versus* Sham** |
| --- | --- | --- | --- | --- | --- |
| **HR, bpm** | **398 ± 66** | **542 ± 20** | **473 ± 17** | 0.184 | 0.042 |
| **CO, mL/min** | **7.6 ± 1.9** | **12.5 ± 2.7** | **14.0 ± 1.0** | 0.007 | 0.001 |
| **ESV, µL** | **59 ± 23** | **14 ± 2** | **13 ± 2** | 0.000 | 0.000 |
| **EDV, µL** | **79 ± 21** | **42 ± 7** | **43 ± 2** | 0.002 | 0.003 |
| **ESP, mmHg** | **65 ± 12** | **70 ± 11** | **76 ± 3** | 1.000 | 0.258 |
| **EDP, mmHg** | **15 ± 7** | **4 ± 9** | **9 ± 6** | 0.107 | 0.605 |
| **Tau, ms** | **16 ± 7** | **14 ± 1** | **13 ± 2** | 1.0000 | 1.000 |
| **dP/dt_MAX_, mmHg/s** | **3774 ± 1615** | **5259 ± 714** | **5664 ± 1141** | 0.231 | 0.090 |
| **-dP/dt_MIN_, mmHg/s** | **3033 ± 1419** | **4239 ± 712** | **4819 ± 961** | 0.301 | 0.065 |
| **SW, mmHg. µL** | **1121 ± 483** | **1951 ± 172** | **2186 ± 333** | 0.009 | 0.001 |
| **EF (%)** | **26 ± 10** | **66 ± 3** | **69 ± 4** | 0.000 | 0.000 |
| ***ESPVR*** |  |  |  |  |  |
| **Slope: E_ES_, mmHg/µL** | **3.83 ± 0.61** | **3.66 ± 1.17** | **3.23 ± 0.34** | 1.000 | 0.769 |
| **Intercept: ESV_INT_, µL** | **63 ± 30** | **20 ± 16** | **11 ± 10** | 0.019 | 0.005 |
| ***EDPVR*** |  |  |  |  |  |
| **Slope: E_ED_, mmHg/µL** | **1.04 ± 0.51** | **0.56 ± 0.22** | **0.43 ± 0.17** | 0.135 | 0.043 |
| **Intercept: EDV_INT_, µL** | **77 ± 22** | **54 ± 25** | **22 ± 52** | 0.951 | 0.094 |

Abbreviations: CO, cardiac output; dP/dt_max_, maximum rate of pressure increase; dP/dt_min_, maximum rate of pressure decrease; EDP, end-diastolic pressure; EDPVR, end-diastolic pressure-volume relationship; E_ed_, end-diastolic stiffness; ; EDV, end-diastolic volume; EDV_int_, end-diastolic volume intercept; EF, ejection fraction; ESP, end-systolic pressure; ESPVR, end-systolic pressure-volume relationship; E_es_, end-systolic elastance; ESV, end-systolic volume; ESV_int_, end-systolic volume intercept; HR, heart rate; SW, stroke work;

Tau, relaxation time constant. N=5 per group. Data are expressed as mean ± SD.
